# Prevention of stone retropulsion during ureteroscopy: Limitations in resources invites revival of old techniques

**DOI:** 10.1080/2090598X.2020.1805966

**Published:** 2020-08-13

**Authors:** Tarek K. Fathelbab, Amr M. Abdelhamid, Ahmed Z.M. Anwar, Ehab M. Galal, Mamdouh M. El-Hawy, Ahmed H. Abdelgawad, Ehab R. Tawfiek

**Affiliations:** School of Medicine, Minia University, Minia, Egypt

**Keywords:** Dormia basket, pneumatic, lithotripsy, stone entrapment, stone retropulsion

## Abstract

**Objective:**

To compare a modified technique using the Dormia basket vs Stone Cone for stone entrapment to avoid proximal stone migration during ureteroscopic pneumatic lithotripsy of ureteric stones.

**Patients and methods:**

Our study included all patients with ureteric stones of <15 mm who underwent ureteroscopic pneumatic lithotripsy from January 2015 to September 2018. The study had two arms that were conducted over two consecutive periods; the first included 72 patients in whom we used the Stone Cone (Group 1) and the second included 86 patients in whom we started to use a Dormia basket with a modification (Group 2) to guard against proximal stone migration.

**Results:**

Both groups were comparable for gender, age, and stone characteristics. Lower ureteric stones were the most prevalent as they represented 62.5% and 60.5% in groups 1 and 2, respectively; while upper ureteric stones were respectively found in 16.7% and 17.4%. Chemical stone analysis revealed that calcium oxalate stones were most predominant accounting for 51.3% and 51.1% in groups 1 and 2, respectively. Most of the stones were radio-opaque stones representing 57% and 58.1% in groups 1 and 2, respectively. There was a significant difference in operative time, with a mean (SD) operative time was 50.9 (11.2) in Group 1 vs 58.3 (12.4) min in Group 2 (*P* < 0.001). The success rate, defined as no retropulsion of stone fragments, was 97.7% in Group 2 vs 91.7% in Group 1 (*P* < 0.01). Complications were minor and comparable between the groups. There was no difference in hospital stay between the groups, but the cost assessment favoured Group 2.

**Conclusion:**

We found that our modified-basket stone entrapment technique compared favourably with the Stone Cone to guard against stone retropulsion during ureteroscopic pneumatic lithotripsy. Our modification to the basket was found to be feasible, efficient, safe, reproducible and cost-effective in preventing proximal stone migration. This procedure is particularly suitable in cost-limited environments.

## Introduction

Ureteroscopic lithotripsy is considered the first-line treatment for ureteric stones that fail to respond to medical expulsive therapy (MET) or shockwave lithotripsy (SWL) [1]. Advances in ureteroscope design and manufacture, as well as stone retrieval devices contribute, to a great extent, in the reported high success rate of ureteroscopic stone extraction [1]. During ureteroscopic lithotripsy, the possibility of stone retropulsion or upward migration limits the success rate. There is a wide variation in the retropulsion rate depending upon the kinetic energy of the lithotripter and ureteric stone level, as proximal stones have a higher rate of stone migration than those that are distally located [1,2].

Migrating stones or fragments may necessitate additional procedures, e.g. flexible ureteroscopy, or secondary procedures such as SWL, with their additional costs; as untreated stone fragments may serve as nidi for new stone growth [2,3].

To guard against this problem, many devices have been used, e.g., the Stone Cone, N-Trap, Back stop, and Accordion. All these devices add extra cost to the procedure [4,5]. Laser lithotripsy is associated with the lowest incidence of stone retropulsion. Because of the widespread use of laser lithotripsy, retropulsion prevention using additional devices has dramatically decreased in many centres. Nevertheless, its high cost has limited its use in countries with modest resources. Many hospitals in different parts of the developing world do not have sufficient resources to cover the price of a laser machine and its running costs. For this reason, we suggest the revival of an old technique of disassembly of a Dormia basket to prevent stone retropulsion during ureteroscopic pneumatic lithotripsy.

In our centre, we used a modified-basket stone entrapment technique to avoid proximal stone migration during ureteroscopic pneumatic lithotripsy. In this way, we could save the extra cost of anti-retropulsion devices and overcome the limited availability of laser lithotripters in centres located in areas with minimal resources.

## Patients and methods

### Study design

This retrospective study included all patients with ureteric stones who underwent ureteroscopic stone removal from January 2015 to September 2018. The study had two arms that were conducted consecutively.

The first arm (Group 1) was performed between January 2015 and January 2016, in which we used the Stone Cone® (Boston Scientific Corp, Natick, MA, USA) to avoid proximal stone migration. Because of some financial difficulties, we could no longer use the Stone Cone; therefore, we used a modification of the stone basket between April 2016 and September 2018 (Group 2). The present study was designed to compare the results of both techniques in the two consecutive periods.

The study comprised 208 patients who underwent ureteroscopic stone removal. Patients with ureteric stones of <15 mm treated by ureteroscopy after failure of MET and/or failure of SWL were included.

Patients with either bilateral (*n* = 10), or multiple (*n* = 24) ureteric stones and patients in whom the stone was extracted directly without disintegration (*n* = 16) were excluded from the study. There were 72 patients treated by Stone Cone (Group 1) and 86 treated by Dormia basket (Group 2). The study was approved by our local Ethics Committee.

### Preoperative assessment

Stone size and location were assessed by non-enhanced multi-detector CT. Routine laboratory tests; urine analysis, culture and sensitivity were performed. All patients received preoperative prophylactic antibiotic. Success was defined as a safely completed procedure with no retropulsion of stone fragments, and no need for any auxiliary manoeuvres. Retropulsion was considered when the stone or fragments migrated upwards and could not be reached by ureteroscopy. Patient demographics, stone criteria, operative time, intraoperative complications, and success rates were reported and statistically analysed.

### Endoscopic procedure

A semi-rigid (8–9.8 F) ureteroscope (Wolf, Knittlingen, Germany) was used in all cases. Stone fragmentation was done using a pneumatic lithoclast (Swiss LithoClast) in all cases.

For Group 1, the Stone Cone was placed under vision via the working channel of the ureteroscope. Once the stone was reached in the ureter, the Stone Cone was opened above the stone and the ureteroscope was re-introduced. Then pneumatic lithotripsy was performed and the fragments were extracted with a Dormia or grasping forceps.

For Group 2, the safety guidewire was advanced under vision beside the stone, the Dormia basket (nitinol basket 3.5–4 F with four wires, Zero Tip™ nitinol basket, Boston Scientific) was used to catch the stone and hold it until it was mildly entrapped. The basket handle was disassembled and detached with the sheath, leaving the stone caught within the wires of the basket. The ureteroscope was then re-introduced. Gentle support was exerted by the operator on the basket core to keep the stone entrapped within the basket. Pneumatic lithotripsy was used while applying gentle and cautious support to the basket. Disintegration was applied to the centre of the stone and continued until we could easily see the tip of basket clear of stones and the basket was retrieved.

Re-assembling of the Dormia was done to allow for retrieval of any disintegrated fragments, with fragments of ≥4 mm considered significant and thus may need further procedures.

At the end of the manoeuvre, retrograde pyelography was done for detection of any complications. A JJ ureteric stent was left *in situ* for 2 weeks in all cases.

All the surgical procedures were done by two senior expert endourologist (E.R.T. and T.KF.)

### Postoperative evaluation

Postoperative follow-up was done using non-enhanced multi-detector CT before the removal of the ureteric stent.

### Statistical analysis

The results are presented as the mean (± SD). Statistical analysis was done using the Statistical Package for the Social Sciences (SPSS®) for Windows, version 11.0 (SPSS Inc., Chicago, IL, USA). The Student’s *t*-test, chi-square test and Fisher’s exact test were used as appropriate. A *P* < 0.05 was considered statistically significant.

## Results

Both groups were comparable for gender, age and stone characteristics, as shown in Table 1. Lower ureteric stones were the most prevalent as they represented 62.5% and 60.5% in groups 1 and 2, respectively, with upper ureteric stones in 16.7% and 17.4%, and mid-ureteric stones in 20.8% and 22.1%. Chemical stone analysis revealed that calcium oxalate stones were most predominant representing 51.3% and 51.1% in groups 1 and 2, respectively. Most of the stones were radio-opaque, 57% in Group1 and 58.1% in Group 2.

**Table 1.** Patients’ demographics and stone data

Variable	Group 1 Stone Cone	Group 2 Dormia	*P*
Number of patients	72	86	
Gender, *n* (%) Male Female	47 (65.2) 25 (34.8)	54 (62.8) 32 (37.2)	0.4
Age, years, mean (SD)	37.6 (11.7)	38.1 (8.9)	0.7
Ureteric stone size, mm, mean (SD)	13.8 (3.5)	13.1 (4.2)	0.1
Stone location, *n* (%) Upper ureter Middle ureter Lower ureter	12 (16.7) 15 (20.8) 45 (62.5)	15 (17.4) 19 (22.1) 52 (60.5)	
Laterality, *n* (%) Right Left	37 (51.3) 35 (48.7)	51 (59.3) 35 (40.7)	0.2
Stone composition, *n* (%) Ca-Oxalate Urate Mixed Stones Triple Phosphate	37 (51.3) 20 (27.8) 7 (9.8) 8 (11.1)	44 (51.1) 24 (27.9) 8 (9.3) 10 (11.7)	0.3
Stone Opacity, *n* (%) Radio-opaque Radiolucent	41 (57) 31 (43)	50 (58.1) 36 (41.9)	0.8

We recorded a significant difference in the operative time in favour of Group 2, at a mean (SD) of 50.9 (11.2) vs 58.3 (12.4) min (*P* < 0.001). The retropulsion rate was significantly different between the groups, at 2.3% in Group 2 vs 8.3% in Group 1 (*P* < 0.01). In Group 1, retropulsion occurred in six patients (four upper and two middle ureteric stones), and after failure of MET, SWL was performed in four and two required flexible ureteroscopy. In Group 2, retropulsion occurred in two patients (one upper and one middle ureteric stone), which were successfully treated by MET.

Minor mucosal abrasion was recorded in 12.5% in Group 1 and 13.9% in Group 2. In Group 2, we recorded four cases in whom wires of the Dormia were inadvertently torn and the Dormia was safely removed under vision without any injury to the ureter and was replaced by a new one. Hospital stay in the present study was comparable between the groups with no significant difference. For cost analysis, there was a cost saving of 240 USD per patient in Group 2, with additional saving for costs of the auxiliary procedures required to treat the stone fragments in Group 1. A summary of overall results is given in Table 2.

**Table 2.** Overall results

Variable	Group 1 Stone Cone (*N* = 72)	Group 2 Dormia (*N* = 86)	*P*
Operative time, min, mean (SD)	50.9 (11.2)	58.3 (12.4)	0.001*
Success rate (no retropulsion), *n* (%)	66 (91.7)	84 (97.7)	0.01**
Complications, *n* (%) Minor laceration Torn wire	9 (12.5) 0	12 (13.9) 4 (4.6)	0.5***
Hospital stay, h, mean (SD)	16.20 (7)	17.67 (4.51)	0.5*

*Student’s *t*-test; **chi-square test; ***Fisher’s exact test.

## Discussion

Ureteroscopy is one of the commonest procedures for treating ureteric stones, with the stone-free rate (SFR) usually >95% and low morbidity [6]. Proximal stone migration may lead to a longer operative time, higher incidence of residual stones and the need for auxiliary procedures, with higher morbidity and greater cost [7]. Pneumatic lithotripters are usually associated with a higher retropulsion rate, while laser has the lowest rate [8]. Several manoeuvres have been described to prevent proximal stone migration, including reverse Trendelenburg position and decreased irrigation pressure and flow rate; however, these techniques, may interfere with surgeon comfort and visibility [9,10].

The use of pneumatic lithotripsy, as well as limited access to flexible ureteroscopes, mandate utilisation of anti-retropulsion devices to achieve higher success rates [11,12].

Many devices have been introduced to minimise the incidence of proximal stone migration during ureteroscopic lithotripsy including Stone Cone, entrapment net (N-Trap), Accordion, BackStop, lidocaine jelly, and thermophilic polymers [8,13].

In the present study, we compared the Stone Cone with Dormia basket stone entrapment to guard against proximal stone migration during ureteroscopic pneumatic lithotripsy. In 2007, Tunc *et al*. [2] used pneumatic lithotripsy for ureteric stone disintegration in 156 patients, with a reported SFR of 85.2% and retropulsion rate of 7.1%. On the other hand, Sözen *et al*. [14] used pneumatic lithotripsy in 500 patients and reported SFR of 95% and migration rate of 2%. Neither of them used anti-retropulsion devices. In the present study, we used a modification of disassembly of a Dormia basket to prevent stone retropulsion during pneumatic lithotripsy. Although this technique may be practiced by many urologists, nevertheless, results of its use have not been properly documented. A similar technique was published by Kesler *et al*. [15] in 2008, the authors used an Escape™ (Boston Scientific) nitinol stone retrieval basket that is a specially designed basket to capture calculi and facilitate simultaneous laser lithotripsy, resulting in a SFR of 87%. However, this device has some limitations as the stone engagement is not handled effectively in many situations, e.g. for impacted stones. Also, this device is more expensive than the traditional nitinol wire baskets, increasing the cost of the procedure.

In the present study, the SFR was 97.7% (84/86) with no requirement for additional procedures using the basket stone entrapment, while the SFR was 91.7% (66/72) when the Stone Cone was used. Nevertheless, Shabana *et al*. [16] in their study using the Stone Cone and N-Trap reported success rates of 97.1% and 95.7%, respectively.

It is known that the Stone Cone specifically acts as a ‘backstop’ and cannot be used for stone removal. Therefore, fragments of <3 mm may escape and this could explain the higher frequency of stone fragments in Group 1 of our present study. The majority of complications during ureteroscopy are minor with reported rates of 0–15.4% [17]. Ureteric perforation and avulsion are major concerns that should be avoided. In the present study, there was minor mucosal abrasion in nine (12.5%) cases in Group 1 and 12 (13.9%) in Group 2, with no reported major ureteric injuries, documented by retrograde pyelography performed at the end of the manoeuvre. Shabana *et al*. [16] reported overall ureteric injuries in 9.2% of cases with ureteric perforation occurring in six (1.4%). Conversely, Desai *et al*. [18] observed minor mucosal abrasion in five (10%) cases, with no major complications.

There is no clear definition of clinically significant residual fragments in the related articles leading to confusion in the reported results [19].

In a study by Desai *et al*. [18], using the Stone Cone as the anti-retropulsion device,12% of cases had residual fragments of >3 mm, but none required any additional manoeuvres; while Shabana *et al*. [16] reported residual fragments in 2.9% and 4.3% of cases in their Stone Cone and N-Trap groups, respectively. In our present study residual fragments were observed only in two cases in Group 2 (2.3%).

An important issue for the stone retropulsion rate is the pressure of the irrigating fluid [10,11]. In our basket entrapment technique the stone is enclosed and entrapped within the Dormia basket, so we can increase the irrigation fluid pressure without fear of proximal escape of the stone allowing good visibility.

Most of the recently used anti-retropulsion devices are safe and efficient during intracorporeal lithotripsy. Each device has its own advantages and disadvantages, but the costs of each device should also be considered [20]. Although these recently developed devices are associated with high success rates, they still add extra cost.

The price of the Stone Cone is 240 USD, while the price of a nitinol Dormia is only 226 USD. As described previously, in Group 1 we had to use both devices, so the additive cost was 466 USD per patient compared to only 226 USD in Group 2. Moreover, auxiliary procedures added further costs for patients in Group 1.

Although current AUA guidelines recommend flexible ureteroscopy to be available for ureteroscopy for proximal ureteric stones, which obviates the need for anti-retropulsion devices, there are a lot of centres in developing countries that do not have flexible ureteroscopy and laser lithotripsy; this makes our technique useful in such situations.

Our technique of using basket stone entrapment is safe, reproducible, easily performed, and is highly effective for all types of stones irrespective of their hardness with no additional cost; an issue of importance especially in the developing countries and for those with limited resources. Limitations of our present study include, the retrospective nature and relatively small sample size. Future randomised prospective studies using this technique are invited to consolidate our present results of its clinical effectiveness and safety.

## Conclusion

Using a re-purposed basket stone entrapment during ureteroscopic pneumatic lithotripsy is feasible, efficient, safe, and cost-effective in preventing proximal stone migration. The procedure deserves special consideration in cost-limited centres.
